# Prevalence of oral rehydration therapy use and associated factors among under-five children with diarrhea in Dangure, Benishangul Gumuz Region, Ethiopia/2018

**DOI:** 10.1186/s13104-019-4078-6

**Published:** 2019-01-30

**Authors:** Haftom Gebrehiwot Misgna, Biratu Ebessa, Mekuria Kassa

**Affiliations:** 1 Department of Pediatrics and Child Health Nursing, School of Nursing, College of Health Sciences, Mekelle University, Mekelle, Ethiopia; 2 Pawi College of Health Sciences, Asosa, Ethiopia

**Keywords:** Diarrhoea, Prevalence, Oral rehydration therapy, Benishangul Gumuz

## Abstract

**Objectives:**

This study aims to assess the prevalence of oral rehydration therapy use and associated factors among under-five children with diarrhoea in Dangure district, Benishangul Gumuz Region, Ethiopia/2018.

**Result:**

A total of 615 under-five children who suffered from diarrhoea 2 weeks before the study were included and the response rate was 610 (99%). Among the total children participated in this study 189 (31%) were between 12 and 23 months with mean 23.5 and SD ± 6.9. Five hundred seventeen (84.8%) of mothers had access to oral rehydration therapy and 85% of mother’s home had taken less than 1 h distance from the health facility. The prevalence of oral rehydration therapy is 51%. Two hundred sixty-seven (43.8%) mothers mentioned correctly about the importance of oral rehydration therapy which is to replace fluid while other 243 (39.8%) mothers stated that oral rehydration therapy uses to decrease diarrhoea. Mother’s educational status, monthly income, knowledge of oral rehydration therapy, previous experience and seeking advice for treatment from health facilities were factors associated with oral rehydration therapy use.

**Electronic supplementary material:**

The online version of this article (10.1186/s13104-019-4078-6) contains supplementary material, which is available to authorized users.

## Introduction

Globally, each year, diarrhoea kills around 7,600,000 children less than 5 years and 1.7 billion cases are reported every year [1]. In Africa, every under-five children experience five episodes of diarrhoea annually, and around 800,000 children die of diarrhoea and dehydration each year [2]. The majority (42%) of these deaths are concentrated in the Sub-Saharan African countries [3]. Diarrhoea kills young children more than acquired immunodeficiency syndrome (AIDS), malaria and measles combined [4]. These children have died because of the previous poor use of ORT by some of the mothers, and these deaths are caused mainly by dehydration which can be treated with ORT [5]. Ethiopia ranks fifth globally as an average 20–27% of child deaths caused by diarrheal diseases [6].

According to the Ethiopian Demographic Health Survey (EDHS) 2016 report in Benishangul Gumuz Regional State, despite the dramatic decrement of under-five mortality rate from 167 to 98 deaths per 1000 lives birth, the prevalence of diarrhoea was 22.1% [7].

The critical factor for the reduction in the mortality from diarrhoea was the introduction of oral rehydration therapy [8, 9]. ORT has now become the mainstay of the World Health Organization (WHO) efforts to decrease diarrhoea morbidity and mortality [10]. Not only does ORT prevent deaths from dehydration, but children had also shown to grow faster and to be better nourished when glucose-based ORT was used [11, 12]. WHO and United Nations Children’s Fund (UNICEF) released a joint statement to decrease diarrheal deaths among the world’s most vulnerable children [13].

A study shows that ORT continues to be underused globally and especially in low-income countries [14]. Analysis of two Demographic and Health Surveys (DHS) conducted in 34 countries showed that 68% of those countries declined in ORT use for children < 5 years of age [15]. In Ethiopia, the use of ORT is only 46% which is far below the recommended [16].

In Ethiopia, estimated thousands of children die every year due to a failure to replace fluid effectively [17]. Community-based cross-sectional study conducted in Assela Town showed that 58.2% of mothers use ORT for their children [18]. Oral rehydration has not yet achieved its full potential to prevent diarrheal deaths due to many factors [19].

### General objectives

This study aims to assess the prevalence of oral rehydration therapy use and associated factors among under-five children with diarrhoea in Dangure district, Benishangul Gumuz Region, Ethiopia in 2018.

### Specific objectives

1. To assess the prevalence of oral rehydration therapy use among under-five children with diarrhoea.
2. To identify the factors associated with oral rehydration therapy use among under-five children with diarrhoea.

## Main text

### Methods

#### Study area and study design

The study was conducted from February to March 2018 in Dangure, a district of Benishagule Gumuz Regional State, North West Ethiopia. The district is located 584 km from Addis Ababa. The health system is represented by three health centres and 27 health posts with 43 Health Extension Workers. In addition to this, there are two medium and thirteen lower clinics owned by private sectors. Community-Based cross-sectional study design was employed. The study populations were under-5 years children who suffered from the diarrheal disease 2 weeks before the study in 14 selected village.

#### Sample size calculation

From the source population, the size of the sample was determined by the following formula.

$${\text{n}} = \frac{{\left( {{\text{Z}}_{{\upalpha/2}} } \right)^{2} {\text{P}}\left( {1 - {\text{P}}} \right)}}{{{\text{d}}^{2} }}$$

where n = minimum sample size required, P = Proportion of ORT use in Assela Town 58.2% in 2015, d = the margin of sampling error tolerated (5%), Z_α/2_ = the confidence interval of 95% (1.96).

Therefore $$n = \frac{{\left( {{\text{Z}}_{{\upalpha/2}} } \right)^{2} {\text{P}}\left( {1 - {\text{P}}} \right)}}{{{\text{d}}^{2} }}$$

$$n = \frac{{\left( {1.96} \right)^{2} \times 0.582 \times 0.418}}{{(0.05)^{2} }}$$

$$n = 373 + 10\% \;{\text{Non-response}}\;{\text{Rate}}$$

$$n = 410*1.5\;{\text{design}}\;{\text{effect}}$$

$$n = 615$$

Based on proportional allocation to size, 615 study participants were distributed to each village.

#### Sampling technique and procedure

Multistage sampling technique was employed to select the study participants. Fourteen villages from 29 were selected randomly by lottery method to ensure representativeness. Each household was selected by systematic sampling techniques. The first household was selected by lottery method and continued every fifth intervals ((K = 3255/615 = 5) where K is the interval value).

#### Data collection tools and procedures

Data was collected using structured and semi-structured interviewer-administered questionnaires from mothers of under-five children [4, 12]. First, the English version of the questionnaire was prepared. Then it was translated to Amharic and back to English. Fourteen diploma holder nurses were selected as data collectors and three other health officers also selected for supervision (Additional file 1).

#### Study variables

*Dependent variables* the outcome variable of the study is the prevalence of oral rehydration therapy use.

#### Independent variable

*Socio*-*demographic characteristic* in this category; Age of child, Number of < 5 years children, Parental educational level, occupation, family size, Age of caregivers, the gender of caregivers, Residence, monthly income, access to ORT and Marital status were included.

*Caregiver behaviour* in this category; knowledge about ORT, previous use of ORT, advice or treatment from health facilities and availability of ORS sachet at home were included.

*Caregivers perceived causes and morbidity of diarrhoea* in this group; caregivers perception on the causes of diarrhoea, number of signs identified to recognise the severity of diarrhoea and dehydration were included.

#### Data processing and analysis

The data were checked manually for completeness, and consistencies then entered into EPI Info-7 for cleaning and transferred to SPSS-20 for analysis. To reduce the excess number of variables in the final model, only those variables with P < 0.25 in the bivariate analysis were considered in the multivariable analysis. Finally, multivariable logistic regression was used to determine predictors of the outcome and to adjust confounding variables. For all statistical tests, P < 0.05 was considered as a cutoff point for statistical significance.

### Results

#### Socio-demographic characteristics

A total of 615 under-five children were included, and the response rate was 610 (99%). Among the participants, 189 (31%) were between 12 and 23 months with a mean of 23.5 and SD ± 6.9. The study indicates that 304 (49.8%) of caregivers had no formal education and 497 (81.5%) of mothers were married (Table 1).

**Table 1:** Socio-demographic factors of participants with ORT usage among under-five children with diarrhoea in Dangure district, Benishangul Gumuze Regional state, Ethiopia, 2018

Variables	Category	Frequency (n = 610)	Percentage (%)
Age of caregiver (years)	15–24	115	18.9
	25–35	364	59.7
	36–45	112	18.4
	> 45	19	3.1
Gender of caregiver	Female	593	97.2
	Male	17	2.8
Age of children in month	0–11	87	14.3
	12–23	189	31
	24–35	171	28
	36–47	104	17
	48–59	59	9.7
Ethnicity	Gumuze	428	70.2
	Shinasha	144	23.6
	Amhara	14	2.3
	Oromo	2	0.3
	Others*	22	3.6
Religion	Orthodox	357	58.8
	Muslim	148	24
	Catholic	64	10.5
	Protestant	28	4.6
	Others*	11	1.8
Residence	Rural	536	87.9
	Urban	74	12.1
Number of under-five children in the household	2 or less	511	83.8
	3 and above	99	16.2
Educational status of mother	No formal education	304	49.8
	Primary school	188	30.8
	Secondary school	90	14.8
	College and above	28	4.6
Occupation caregiver	Farmer	380	62.3
	Housewife	184	30.2
	Merchant	32	5.2
	Govt. employee	14	2.3
Marital status	Married	497	81.5
	Divorced	68	11.2
	Widowed	35	5.7
	Single	10	1.6
Access to ORS	Yes	517	84.8
	No	93	15.2

Religion (Others*)—Adventist; Ethnicity (Others*)—Agew, Kambata

Five hundred seventeen (84.8%) of mothers had access to ORT, and 85% of mothers’ home had less than 1 h distance from the health facility. Among the study participants, 273 (44.8%) of the caregivers have a good knowledge of ORT utilization.

#### Prevalence of ORT utilization among under-five children with diarrhoea

According to this study 235 (38.5%) of mothers heard about ORT from HEWs while, 193 (31.6%), 119 (19.5%) and 63 (10.4%) heard from the health centre, friends/relatives and radio, respectively. This result shows that 311 (51%) of caregivers administered oral rehydration therapy for the management of diarrhoea. Two hundred sixty-seven (43.8%) caregivers had mentioned correctly that ORT is essential to replace fluid loss while other 243 (39.8%) caregivers stated that ORT uses for decreasing diarrhoea. Among the total 538 mothers who knew about the importance of ORT, 257 (48%) caregivers had used an ORT prepared within 1 day duration while, 175 (32%), 62 (11.5%) and 44 (8.5%) had used an ORT prepared within the second, third and fourth day duration, respectively. This study showed that 330 (54.1%) of mothers answered correctly about the preparation of ORT.

#### Factors associated with ORT utilization of mothers/caregivers on bivariate analysis

Mothers who had higher income were 1.9 times more likely to use ORT compared to those who had lower income [COR (95% CI) (1.3–2.7)]. Four hundred thirteen (67.7%) of caregivers identified only one sign of dehydration correctly [COR = 2.1, 95% CI (1.4–3.16)] (Table 2).

**Table 2:** Association of the mother’s perceived causes and morbidity of diarrhoea with ORT usage among under-five children in Dangure district, Benishangul Gumuze region, Ethiopia, 2018

Variables	Category	Yes	No	COR (95% CI)	P-value
ORT use					
Perceived cause of diarrhoea					
Tooth eruption	No	133 (36.2%)	234 (63.8%)	1	
	Yes	178 (73.3%)	65 (26.7%)	4.81 (4.60–5.00)	0.000
Contaminated food	No	251 (57.4%)	186 (42.6%)	1	
	Yes	60 (34.7)	113 (65.3%)	0.39 (0.27–0.56)	0.032
Number of signs identified to recognise the severity of diarrhoea	Non	39 (35.5%)	71 (64.5%)	1	
	1	221 (52.4%)	201 (47.6%)	2.0 (1.29–3.09)	0.002
	≥ 2	51 (65.4%)	27 (34.6%)	3.4 (1.87–6.31)	0.001
Number of signs identified to recognise dehydration	Non	58 (38.2%)	94 (61.8%)	1	
	1	236 (57.1%)	177 (42.9%)	2.1 (1.4–3.16)	0.015
	≥ 2	17 (37.8%)	28 (62.2%)	0.98 (0.49–1.95)	0.963

#### Factors associated with ORT use towards the management of diarrhoea on multivariate analysis

Mothers who had good Knowledge about ORT were 1.5 times more likely to use ORT compared to those who had poor Knowledge [AOR (95% CI) (1.14–3.90)]. Mothers who attended primary school were 2.8 times more likely to use ORT compared to those who had no formal education [AOR (95% CI) (1.52–5.33)]. Similarly, mothers’ who had previous experience of ORT use were 8.5 times more likely to use ORT compared to those who had no previous experience [AOR (95% CI) (5.20–15.1)]. Caregivers who perceived teething as the cause of diarrhoea, 76% of them were more likely to use ORT than their counterparts [AOR (95% CI) 0.24 (0.65–0.98)] (Table 3).

**Table 3:** Factors associated with ORT utilisation of mothers towards management of diarrhoea for under-five children in Dangure district, Benishangul Gumuze region, Ethiopia/2018

Variables	Yes	No	AOR (95% CI)	P-value
Knowledge about ORT				
Good	147 (53.8%)	126 (46.2%)	1.5 (1.14–3.90)	0.038
Poor	164 (48.7%)	173 (51.3%)	1	–
Maternal educational status				
No formal education	130 (42.8%)	174 (57.2%)	1	–
Primary school	108 (57.4%)	80 (42.6%)	2.8 (1.52–9.33)	0.001
Secondary school	54 (60%)	36 (40%)	3.2 (1.69–6.14)	0.002
College and above	19 (67.9%)	9 (32.1%)	4.1 (1.5–10.9)	0.004
Monthly income				
< 5000 birr	72 (36.4%)	126 (63.6%)	1	–
500–1000 birr	131 (52.2%)	120 (47.8%)	2.1 (1.14–3.25)	0.014
1001–3000 birr	71 (63.4%)	41 (36.6%)	2.8 (1.51–6.24)	0.002
> 3000 birr	37 (75.5%)	12 (24.5%)	4.3 (1.78–12.5)	0.012
Seeking advice or treatment from health facility				
Yes	251 (58.2%)	180 (41.8%)	3.9 (2.90–8.49)	0.020
No	60 (33.5%)	119 (66.5%)	1	–
Previous experience of ORT				
Yes	265 (68.5%)	122 (31.5%)	8.5 (5.20–15.1)	0.031
No	46 (20.6%)	177 (79.4%)	1	–
Had ORT sachet at home				
Yes	63 (72.4%)	24 (27.6%)	2.7 (1.39–5.25)	0.003
No	248 (47.4%)	275 (52.6%)	1	–
Tooth eruption				
Yes	133 (36.2%)	234 (63.8%)	0.24 (0.6–0.98)	0.000
No	178 (73.3%)	65 (26.7%)	1	–

### Discussion

In this study, the prevalence of ORT use was 51% [95% CI (47.02–54.93)]. This result is lower than previous studies in which 61.8% in a Military Barrack in Ibadan, Nigeria [14], 61% in Kenya [9] and 58.2% in Assela Town [18]. This might be due to socio-demographic differences among study participants, and caregivers who were living in the town have more access and opportunity for information about ORT. However, it was higher compared with a study done in Kano State, Nigeria which is 37.6% [10] and 34.6% in Western China [11]. This difference might be due to a long-standing source of speculation in which caregivers need parental preference of treatment.

In this study, mothers who were with a good knowledge about ORT were 1.5 times more likely to use ORT than their counterparts [AOR (95% CI (1.14–3.90)] which is lower associated than a study conducted in Finote Selam [20] (AOR = 15.46). This difference might be due to the low level of education and awareness of the mothers on diarrhoea management. This is in line with a study conducted in Kerisa district [4] (AOR = 3.09). This similarity might be due to mothers’ perception about advice/information on ORT from health workers.

Mothers who attended primary school were 2.8 times more likely to use ORT compared to those who had no formal education [AOR (95% CI (1.52–9.33)]. It is in line with the studies done in Finote Selam Town [20] (AOR = 3.34), in Ginchi Town [21] (AOR = 8.41) and Fagita of Lekoma District [22] (AOR = 1.63). This might be due to similar socio-demographic characteristics and the economic status of participants. However, this is not associated with studies conducted in Kerisa district [4] (AOR = 1.03). This difference might be due to the social class difference.

Mothers who had 500–1000 Birr income were 2.18 times more likely to use ORT than those who are under 500 Birr income [AOR (95% CI) (1.14–3.25)]. It is in line with a study conducted in Ginchi [21] (AOR = 1.15). This might be due to the similar socio-economic status of the study participants. However, the finding is not in line with a study conducted in the urban areas of India [19] (AOR = 1.07) This might be due to: difference in cultural and social beliefs. Mothers who were seeking treatment from health facilities were 3.9 times more likely to use ORT than their counterparts [AOR 95% CI (2.90–8.49)]. This finding is consistent with studies conducted in Western Kenya [15] (AOR = 3.90), Kerisa district [4] (AOR = 3.25) and associated with studies conducted in the Assosa district [23]. This similarity might be due to the awareness of diarrhoea management among participants and health care service related situations.

According to this study, caregivers who had previous experiences of ORT were 8.5 times more likely to use ORT than their counterparts [AOR = 95% CI (5.20–14.1)]. This finding is in agreement with a study conducted in Kerisa district [4] (AOR = 4.05). This may be explained by the fact that familiarity with ORT could be gained through experience. Caregivers who gave ORT for their children at any time in the past were more likely to use ORT. But not associated with the study conducted in India [24] (AOR = 3.24). This difference might be due to mothers’ having low prior exposure to ORT, lacks awareness about ORT and cultural difference.

The result of this study will be helpful to increase the ORT uptake by promoting maternal educational status and knowledge about the perfect mixture, function, and appropriate quantity of ORT administration [25].

## Limitations

This study was done retrospectively which might cause recall bias due to failure of the caregiver to remember what was happened previously. In addition to this, the study design also may affect the real relationship of the exposure and outcome. It is known that cross-sectional studies could not show the time-effect relationship because it does not tell whether the exposure or the outcome happens first.

## Additional file

**Additional file 1.** English version questionnaires.

## Abbreviations

AIDS: acquired immunodeficiency syndrome
AOR: adjusted odds ratio
CIMCH: Community Integrated Management of Childhood Illnesses
COR: crude odds ratio
DHS: Demographic and Health Surveys
EDHS: Ethiopian Demographic Health Survey
HEW: Health Extension Workers
ORT: oral rehydration therapy
SPSS: Statistical Package for Social Science
UNICEF: United Nations Children’s Fund
WHO: World Health Organization

## References

[1] Forsberg BC, Sreeramareddy CT, Low Y-P (2017). Slow progress in diarrhoea case management in low and middle-income countries: evidence from cross-sectional national surveys, 1985–2012. BMC Paediatrics.

[2] World Health Organization (2009). Diarrhoea: why children are still dying and what can be done.

[3] Mohammed S, Tamiru D (2014). The burden of diarrheal diseases among children under five years of age in Arba Minch District, Southern Ethiopia, and associated risk factors: a cross-sectional study. Int Sch Res Not.

[4] Mengistie B, Berhane Y, Worku A (2012). Predictors of oral rehydration therapy use among under-five children with diarrhoea in Eastern Ethiopia: a community-based case-control study. BMC Public Health.

[5] Kadam D, Hadaye R, Pandit D (2012). Knowledge and practices regarding oral rehydration therapy among mothers in a rural area of Vasind, India. Nepal Med Coll J.

[6] Black RE, Cousens S, Johnson HL, Joy EL, Igor R, Diego GB, Prabhat J, Harry CFC, Richard C, Thomas E, Li L, Colin M (2010). Global, regional, and national causes of child mortality in 2008. A systematic analysis. Lancet..

[7] Mihrete TS, Alemie GA, Teferra AS (2014). Determinants of childhood diarrhoea among under five children in Benishangul Gumuz Regional State, North West Ethiopia. BMC Paediatrics.

[8] Munos MK, Walker CLF, Black RE (2010). The effect of oral rehydration solution and recommended home fluids on diarrhoea mortality. Int J Epidemiol..

[9] Zwisler G, Simpson E, Moodley M (2013). Treatment of diarrhoea in young children: results from surveys on the perception and use of oral rehydration solutions, antibiotics, and other therapies in India and Kenya. J Glob Health.

[10] Bello UL (2015). Comparative Studies of knowledge and perception of parents on home management of diarrheal diseases among under-five children between two communities of Kano State, Nigeria. Int J Pharm Sci Invent..

[11] Gao W (2013). Oral rehydration salt use and its correlates in low-level care of diarrhoea among children under 36 months old in rural Western China. BMC Public Health.

[12] Ema S (2016). Utilization of oral rehydration therapy in the management of diarrhoea in children among nursing mothers in Odukpani local government area of Cross River State, Nigeria. Am J Public Health Res.

[13] Diallo AF. Evaluation of the therapeutic management of childhood diarrhea. 2016.

[14] Agbolade M, Dipeolu I, Ajuwon A (2015). Knowledge and use of oral rehydration therapy among mothers of under-five children in a Military Barrack in Ibadan, Nigeria. Afr J Biomed Res.

[15] Olson CK (2011). Community case management of childhood diarrhoea in a setting with declining use of oral rehydration therapy: findings from cross-sectional studies among primary household caregivers, Kenya, 2007. Am J Trop Med Hyg.

[16] Demographic E (2016). Health survey 2016 central statistical agency Addis Ababa.

[17] Houston KA, Gibb JG, Maitland K (2017). ral rehydration of malnourished children with diarrhoea and dehydration: a systematic review. Wellcome Open Res.

[18] Eshete A (2015). Assessment of knowledge, practice and utilization of oral rehydration therapy for acute watery diarrhoeal disease case management among mothers (caregivers’) of under-five children in Assela town, Ethiopia.

[19] Saurabh S (2014). Knowledge and practice regarding oral rehydration therapy for acute diarrhoea among mothers of under-five children in an urban area of Puducherry India. Natl J Community Med.

[20] Amare D (2014). Maternal knowledge and practice towards diarrhoea management in under five children in Finote Selam Town, West Gojjam Zone, Amhara Regional State, Northwest Ethiopia, 2014. J Infect Dis Ther.

[21] Bekle G. Mothers’/caregivers’ knowledge, attitude and practice about management of diarrhoea and associated factors in under five children in Ginchi town, West Shawa, Oromia region, Ethiopia. 2017.

[22] Desta BK, Assimamaw NT, Ashenafi TD (2017). Knowledge, practice, and associated factors of home-based management of diarrhea among caregivers of children attending under-five clinic in Fagita Lekoma District, Awi Zone, Amhara Regional State, Northwest Ethiopia, 2016. Nurs Res Pract.

[23] Merga N, Alemayehu T (2015). Knowledge, perception, and management skills of mothers with under-five children about the diarrhoeal disease in indigenous and resettlement communities in Assosa District, Western Ethiopia. J Health Popul Nutr.

[24] Gazi E (2015). Can mothers care for the acute diarrhoeal disease of their under-five children effectively at home? A cross-sectional study in a slum community in Bankura. J Evid Based Med Healthcare.

[25] Onwukwe Sergius, van Deventer Claire, Omole Olu (2016). Evaluation of the use of oral rehydration therapy in the management of diarrhoea among children under 5: knowledge attitudes and practices of mothers/caregivers. South Afr Fam Pract.

