# Supplementary material for: Prevalence of oral rehydration therapy use and associated factors among under-five children with diarrhea in Dangure, Benishangul Gumuz Region, Ethiopia/2018
Source: BMC Res Notes. 2019 Jan 30;12:67. doi: 10.1186/s13104-019-4078-6 (PMC6354353; doi:10.1186/s13104-019-4078-6)
Supplement: Supplementary file 1 — Additional file 1. English version questionnaires. [file 13104_2019_4078_MOESM1_ESM.pdf]

# English Version Questionnaires

| S.No | Question | Option | Remark |
|---|---|---|---|
| 101 | Age of caregiver ( in years) | ------------------------ | |
| 102 | Sex of caregivers | 1. Male<br>2. Female | |
| 103 | Religion | 1.Orthodox<br>2. Muslim<br>3. Catholic<br>4. Protestant<br>5. Other (specify-------- | |
| 104 | Ethnicity | 1.Gumuze<br>2.Shinasha<br>3.Amhara<br>4.Oromo<br>5. Others……. | |
| 105 | Female caregivers educational level | 1.No formal education<br>2.Primary school<br>3.Secondary school<br>4. College and above | |
| 106 | Male caregivers educational level | 1.No formal education<br>2.Primary school<br>3.Secondary school<br>4. College and above | |
| 107 | Occupation of female caregivers | 1. House wife<br>2. farmer<br>3. Governmental employee<br>4. Merchant<br>5. Other specify….. | |
| 108 | Relation of caregiver to client | 1. Mother<br>2. Father<br>3. Sibling<br>4. Grand mother<br>5. Grand father<br>6. Other, specify ….. | |
| 109 | Family size | ----------------------- | |
| 110 | Monthly income | -------------------------- | |
| 111 | Residence | 1. Rural<br>2. Urban | |

| | | | |
|---|---|---|---|
| 112 | Marital status | 1a. Single<br>2. Married<br>3. Divorced<br>4. Widowed | |
| 113 | If married, what does your Husband/ wife do? | 1. Governmental employee<br>2. farmer<br>3. Merchant,<br>4. Others | |
| 114 | How old is your child (in months)? | ------------------ | |
| 115 | How many children under five do you have in this house hold? | ------------------------- | |
| 116 | Distance of home from health facility in hour? | 1. Less than 1 hour<br>2.1 hour and above | |
| 117 | From where do you heard about ORT? | 1.Health Extension Worker<br>2. Health center<br>3. Radio<br>4. Friends / relatives<br>5. Other specify ……. | |
| 118 | Do you know the importance of giving ORT to your child? | 1. To increase the diarrhea<br>2. To decrease the diarrhea<br>3. To prevent dehydration<br>4. No idea | |
| 119 | Do you know when to start ORT? | 1. Yes<br>2. No | |
| 120 | How do you prepare ORS at home? | 1. 1 sachet of ORS -300 mls ( 1 coke bottle) of water<br>2. 1 sachet of ORS -500 mls<br>( 1 small size of mineral bottle) of<br>water<br>3.1 sachet of ORS -600 | |

| | | | |
|---|---|---|---|
| | | mls<br>( 2 coke bottle) of water<br>4. 1 sachet of ORS - 1000mls ( 1 liter) of water<br>5.1 sachet of ORS - 1500mls (1.5 liter) of water<br>6. 1 sachet of ORS - 2000mls ( 2 liters or large size of mineral bottle) of water | |
| 121 | When do you use the prepared ORS? | 1. within 24 hrs. ( 1 day)<br>2. within 48 hrs. (2 days)<br>3. within 72 hrs. (3 days)<br>4. within 96 hrs. (4 days)<br>5. Other, specify | |
| 122 | Do you have access to ORS? | 1. Yes<br>2. No | |
| 123 | Did you give recommended Home-made fluid to a child with diarrhea? | 1.Yes<br>2. No | |
| 124 | If yes,what available recommended Home-made fluid / home base Oral Rehydration fluid can be given to a child with diarrhea? ( Tick as many as possible) | 1.Sugar with water<br>2. Salt with water<br>3. Rice water<br>4. Soup<br>5. not used<br>6. Other, specify……… | |
| 125 | Do you have previous experience in using ORT | 1. Yes<br>2. No | |
| 126 | Do you have behavior of seeking treatment from health facilities | 1. Yes<br>2. No | |
| 127 | Do you have ORS sachet at home? | 1. Yes<br>2. No | |

| | | | |
|---|---|---|---|
| 128 | What do you think are the cause of Diarrhea? ( Tick as many as possible) | 1.Tooth eruption<br>2.Evil Eye<br>3.Weaning<br>4.contaminated food/water<br>5.No idea about the cause | |
| 129 | What are the numbers of signs identified to recognize the severity of diarrhea? | 1. fever,<br>2 .repeated vomiting<br>3. many watery stools<br>4. not able to eat or take fluid,<br>5. blood in the stool<br>6 non | |
| 130 | What are the numbers of signs identified to recognize dehydration | 1. excessive thrust,<br>2. sunken eye<br>3. reduced urine output<br>4. poor skin turgor<br>5. restlessness<br>6. Other specify…… | |
